# Comparative proteomic analyses reveal the proteome response to short-term drought in Italian ryegrass (*Lolium multiflorum*)

**DOI:** 10.1371/journal.pone.0184289

**Published:** 2017-09-14

**Authors:** Ling Pan, Zhongfu Yang, Jianping Wang, Pengxi Wang, Xiao Ma, Meiliang Zhou, Ji Li, Nie Gang, Guangyan Feng, Junming Zhao, Xinquan Zhang

**Affiliations:** 1 Department of Grassland Science, College of Animal Science and Technology, Sichuan Agricultural University, Chengdu, China; 2 Agronomy Department, University of Florida, Gainesville, FL, United States of America; 3 Biotechnology Research Institute, Chinese Academy of Agricultural Sciences, Beijing, China; Huazhong Agriculture University, CHINA

## Abstract

Drought is a major abiotic stress that impairs growth and productivity of Italian ryegrass. Comparative analysis of drought responsive proteins will provide insight into molecular mechanism in *Lolium multiflorum* drought tolerance. Using the iTRAQ-based approach, proteomic changes in tolerant and susceptible lines were examined in response to drought condition. A total of 950 differentially accumulated proteins was found to be involved in carbohydrate metabolism, amino acid metabolism, biosynthesis of secondary metabolites, and signal transduction pathway, such as β-D-xylosidase, β-D-glucan glucohydrolase, glycerate dehydrogenase, Cobalamin-independent methionine synthase, glutamine synthetase 1a, Farnesyl pyrophosphate synthase, diacylglycerol, and inositol 1, 4, 5-trisphosphate, which might contributed to enhance drought tolerance or adaption in *Lolium multiflorum*. Interestingly, the two specific metabolic pathways, arachidonic acid and inositol phosphate metabolism including differentially accumulated proteins, were observed only in the tolerant lines. Cysteine protease cathepsin B, Cysteine proteinase, lipid transfer protein and Aquaporin were observed as drought-regulated proteins participating in hydrolysis and transmembrane transport. The activities of phospholipid hydroperoxide glutathione peroxidase, peroxiredoxin, dehydroascorbate reductase, peroxisomal ascorbate peroxidase and monodehydroascorbate reductase associated with alleviating the accumulation of reactive oxygen species in stress inducing environments. Our results showed that drought-responsive proteins were closely related to metabolic processes including signal transduction, antioxidant defenses, hydrolysis, and transmembrane transport.

## Introduction

Drought is the largest abiotic stress factor leading to reduce the productivity of Italian ryegrass, especially at the seedling stage. The effect of drought stress on plants is usually characterized by reduced leaf water content, decreased cell growth, and inducted oxidative stress [1, 2]. The ability of plants to acclimate to such conditions through appropriate regulation is a key determinant of their survival [3]. Recent research has clearly demonstrated that stress responses rely on the functioning of complex gene networks. When plants regularly experience drought, extensive modification of gene accumulation occurs and results in alterations in protein synthesis (up- or down-regulation) [4]. These proteins react to stress by regulating metabolic homeostasis and detoxifying harmful elements such as reactive oxygen species (ROS) [5, 6]. Hence, identification of responsive proteins involved in drought tolerance is a major interest to plant scientists.

Early proteome analyses used two-dimensional gel electrophoresis for protein separation and tandem mass spectrometry for protein identification [7]. Out of a total of 455 proteins identified, 17 differentially accumulated proteins existed in *L*. *multiflorum* and *F*. *arundinacea* introgression lines [8]. With the rapid innovations in proteomics new methods have been developed for protein analysis. The iTRAQ (Isobaric tags for relative and absolute quantification) is a quantitative proteomic method for examining multiple samples in a single mass analysis, thereby enable sensitive assessment and quantification of protein levels [9, 10]. Based on the iTRAQ approach, by comparing tolerant and susceptible cultivars, many proteins were discovered that had the potential to enhance resistance in plants [11, 12].

Italian ryegrass (*Lolium multiflorum* L.) is one of most widespread cultivated cool-season forage grass in the world. Typically, it is grown in a mixture with other grass and legume species to improve pasture quality [13]. In southern China, *L*. *multiflorum* is most commonly served as an annual forage crop for feeding [14]. Although Italian ryegrass expresses some levels of drought tolerance, it still suffers a significant reduction of yield under drought conditions [15], and does not match *F*. *arundinacea* with respect to the potential of tolerance [16]. This potential, however, can be significantly improved in intergeneric *L*. *multiflorum* x *F*. *arundinacea* hybrids, and their introgression derivatives [8, 17].

However, there have been few reports on the regulatory mechanisms of drought tolerance at proteome level for Italian ryegrass. By using the iTRAQ-based method, two *L*. *multiflorum* lines, drought-tolerant “Abundant 10” and drought susceptible “Adrenalin 11” were used in the study to evaluate differentially accumulated proteins under drought stress. This study provides a novel proteomic data for further dissection the regulatory mechanisms of drought tolerance in *Lolium multiflorum* response to short-term drought.

## Materials and methods

### Plant materials and drought treatments

Two *L*. *multiflorum* lines, drought-tolerant “Abundant 10” and drought susceptible “Adrenalin 11” were used in this study [18]. Seeds were germinated on filter paper moistened with distilled water in an environment kept at 25°C.Seedlings were then transferred into plastic pots filled with the Hoagland’s nutrient solution and put into growth chambers with a 16/8 hour day-night cycle, a 25/18°C day-night temperature, and relative humidity of 60%.

One half of the 20-day-old seedlings of the two *L*. *Multiflorum* lines were grown in aerated hydroponics containing Hoagland’s nutrient solution at 25°C to be used as the control and the remaining seedlings were treated under drought stress condition, by lying on plastic trays and naturally air-drying for 2 hours at 25°C in the growth chamber. Ten individual plants for each *L*. *Multiflorum* line were used as biological replicate. We performed two biological replicates for each treatment in the experiment (S1 Fig). Therefore, 20 drought tolerant seedlings under control, 20 drought tolerant seedlings with drought stress treatment, 20 drought susceptible seedlings under control, and 20 drought susceptible seedlings with drought stress treatment, were immediately frozen in liquid nitrogen, and stored at -80°C until protein extraction.

### Measurement of antioxidant activity

Three biological replicates were used for each treatment. Hydrogen peroxide content (H_2_O_2_), phospholipid hydroperoxide glutathione peroxidase (PHGPx), peroxiredoxin (Prx) and ascorbate peroxidase (APX) activities were assayed separately by hydrogen peroxide, PHGPx, Prx and APX assay kits (Comin Biotechnology Co., Ltd. Suzhou, China) according the manufacturer’s menu. Statistical analysis was performed with one-way ANOVA in SPSS 20.0 and all the data were average means of three independent experiments ± SDs.

### Protein extraction

Total proteins of Italian ryegrass samples were extracted with Lysis Buffer 3 containing 1 mM PMSF and 2 mM EDTA, and suspended at 200 W for 15 min. Proteins were isolated by centrifuging at 30000×g for 15 min at 4°C, and were added 5× volume of chilled acetone and 10% (v/v) TCA at -20°C. After two rounds of centrifugation, the supernatant was carefully discarded and the precipitate was washed three times with cold acetone. The protein pellet was air-dried by lyophilization and dissolved in Lysis buffer (7 M urea, 2 M thiourea, 4% NP40, 20 mM Tris-HCl, pH 8.0–8.5). The protein pellet was suspended for 15 min and centrifuged at 4°C at 25000×g for 15 min, and the supernatant was collected. To reduce disulfide bonds in the proteins of the supernatant, 10 mM DTT was added and left at 56°C for 1 hr. Subsequently, 55 mM IAM was added to block the cysteines, so samples were kept in a darkened room for one hour. The supernatant of proteins were kept at -80°C.

### iTRAQ labeling and SCX fractionation

Protein samples of 100 μg each was added to 2.5μg Trypsin (Promega, Madison, WI, USA) with a weight ratio of 40 protein: 1 trypsin and kept at 37°C for 4 hr. The peptides were dried using vacuum by Strata X and were reconstituted in 0.5 M TEAB based on the manufacture’s protocol for 8-plex iTRAQ reagent (Applied Biosystems). This included one unit of thawed and reconstituted iTRAQ reagent in 24 μL isopropanol. The peptides were labeled with isobaric tag, which were pooled and dried through vacuum.

SCX chromatography was performed using a LC-20AB HPLC Pump System (Shimadzu, Kyoto, Japan). The iTRAQ-labeled peptide mixtures were reconstituted with 2 mL buffer A (5% ACN, pH 9.8) and loaded onto a 5 um 4.6×250 mm Ultremex SCX column (Phenomenex, USA). The peptides were eluted at a flow rate of 1 mL/min with a gradient of buffers as following: 5% buffer B (95% ACN, pH 9.8) for 10 min, 5–35% buffer B for 40 min. 35–95% buffer B for 1 min. The system was then maintained in buffer B for 3 min before equilibrating with buffer B for 10 min. Elution was monitored by measuring the absorbance at 214 nm, and fractions were collected every 1 min. The eluted peptides were pooled into 20 fractions and desalted.

### LC–ESI-MS/MS analysis using the Triple TOF 5600 System

Each fraction was re-suspended in buffer A (2% ACN, 0.1% FA) and centrifuged at 20000 g for 10 min, the final concentration of peptides was approximately 0.5 μg/μL on average. 10 μL supernatant was determined by a LC-20AD Nano-HPLC (Shimadzu, Kyoto, Japan) with an autosampler and the peptides were eluted onto analytical C18 column (inner diameter 75 μm and column length15 cm). The samples were loaded for 4 min, then gradient run from 5% buffer B (96% ACN, 0.1% FA) for 0–8 min, linear gradient to35% B for 8–43 min, keep at 60% B for 43–48 min, and return to 5% B for 55-65min.

Data acquisition was performed using a TripleTOF 5600 System (SCIEX, Framingham, MA, USA) fitted with a Nanospray III source (SCIEX, Framingham, MA,USA) and a pulled quartz tip as the emitter (New Objectives, Woburn, MA, USA). Data was acquired using an ion spray with 2.5 kV voltage and, curtain gas was set at 30 psi, nebulizer gas was set at 15 psi, and the interface heater temperature was 150°C. The MS was operated by a resolving power (RP) of 30,000FWHM for TOF/MS scans. Survey scans were obtained from 250 ms and up to 30 product ion scans (cut-off threshold was 120 counts per second (counts/s). Raw data files were transformed into MGF files using Proteome Discoverer software.

### Protein identification and data analysis

The Mascot 2.3.02 search engine (Matrix Science, London, UK; version 2.3.02) was used to identify and quantify proteins. To identify proteins the following parameters were set: 1) a mass tolerance of 2 Da (ppm) was permitted for intact peptide masses, 2) the Peptides matching error was set at 0.05 Da, 3) Gln- > pyro-Glu (N-term Q), Oxidation (M), Deamidated (NQ) were set as potential variable modifications, 4) Carbamidomethyl (C), iTRAQ8plex (N-term), iTRAQ8plex (K) were set as fixed modifications, and 5) the charge states of the peptides were set to +2 and +3. An automatic decoy database search can be performed by choosing the decoy checkbox to produce a random sequence database and to test for raw spectra.

A 95% confidence interval was used to identify peptide using the Mascot probability analysis. Each protein was identified by at least one unique peptide and each protein should contain at least two unique spectra. The protein sequence database (NCBInr (http://www.ncbi.nlm.nih.gov), SwissProt (http://www.ebi.ac.uk/swissprot), and UniProt (http://www.uniprot.org) was used for the protein identifications. The quantitative protein ratios were measured and normalized in Mascot. A 2-fold change, statistical P-values < 0.05 and false discovery rate (FDR) < = 0.01 were used as criteria for identifying differentially accumulated proteins. Functional annotations of identified proteins were performed using Blast2GO against the Non-redundant protein database (NR). The KEGG database and the clusters of orthologous groups (COG) database were applied to classify the identified proteins. The data including the number of identified peptides, peptides masses, peptides sequence, and peptides scores were provided in S1 Table.

### Western blotting

For each protein sample 10 ug was loaded on SDS PAGE gel (1.5 mm). Migration of proteins in the PAGE gel was conducted at 150 V until the blue band from the sample buffer run out of the gel. Protein-Marker IV was also loaded to determine the molecular weight of the proteins, Proteins were then transferred onto a Polyvinylidene fluoride (PVDF) membrane (Millipore, USA). The following antibodies were used in the western blot analysis: actin (ACT), ADP-glucose pyrophosphorylase (ADGP), β-amylase, isocitrate lyase (ICL), aquaporin, plasma membrane intrinistic protein 1–3 (PIP1), tonoplast intrinsic protein 1-1(TIP1), heat shock protein 90 (HSP90), dehydroascorbate reductase (DHAR1), and alpha-amylase (from Agrisera, Sweden). The PVDF membrane was probed with primary antibody and developed using enhanced chemilu-minescence detection (PerkinElmer, Waltham MA, USA). The blots were detected using the BeyoECL plus (P0018). The images were obtained with the ChemiDoc ^TM^ MP imaging system, and the quantifications were conducted with the software Image Lab ^TM^ V5.1.

## Results

### Changes in activities of antioxidative enzymes in drought treated *L*. *multiflorum*

**Fig 1:**
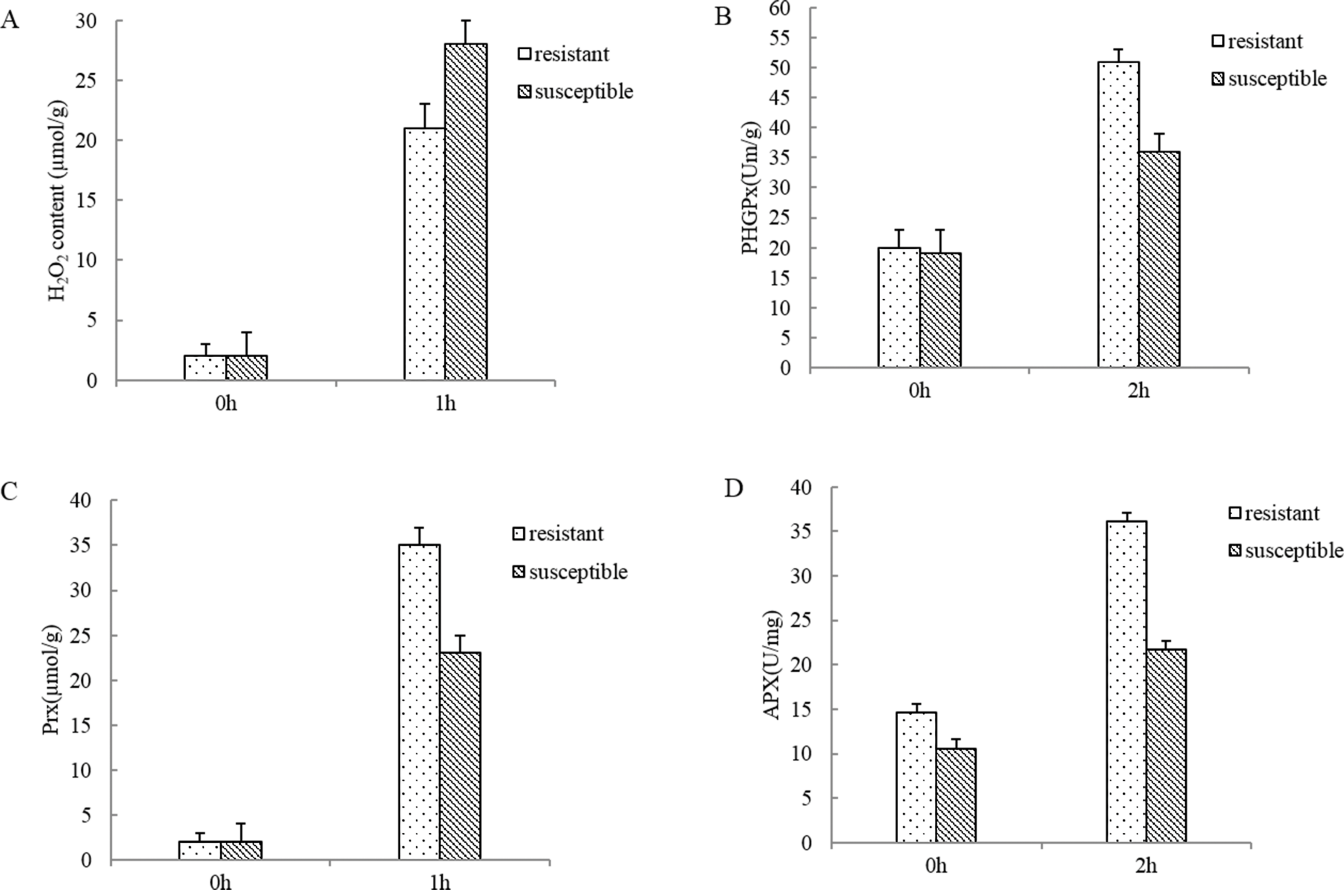
Changes in H_2_O_2_ content and enzyme activities of hospholipid hydroperoxide glutathione peroxidase (PHGPx), peroxiredoxin (Prx) and ascorbate peroxidase during drought stress in the two *L*. *multiflorum* lines. The different letters above the columns indicate significant differences between different time points (P<0.05).

To investigate the effects of drought stress on the oxidative stress, the two *L*. *multiflorum* lines under control and drought treatment were tested for H_2_O_2_ content, enzyme activities of PHGPx, Prx and APX. Under drought stress, H_2_O_2_ content in both *L*. *multiflorum* lines showed dramatically increase compared with control seedlings (Fig 1A), especially in susceptible. A higher enzymatic activities of hospholipid PHGPx, Prx and APX were observed in the tolerant lines exposed to drought stress than under control (Fig 1B–1D). These results indicated that antioxidant enzymes contributed to alleviated oxidative stress- triggered ROS accumulation in tolerant lines during short-term drought stress treatment.

### Differential accumulation analysis of *L*. *multiflorum* proteome under drought stress

**Fig 2:**
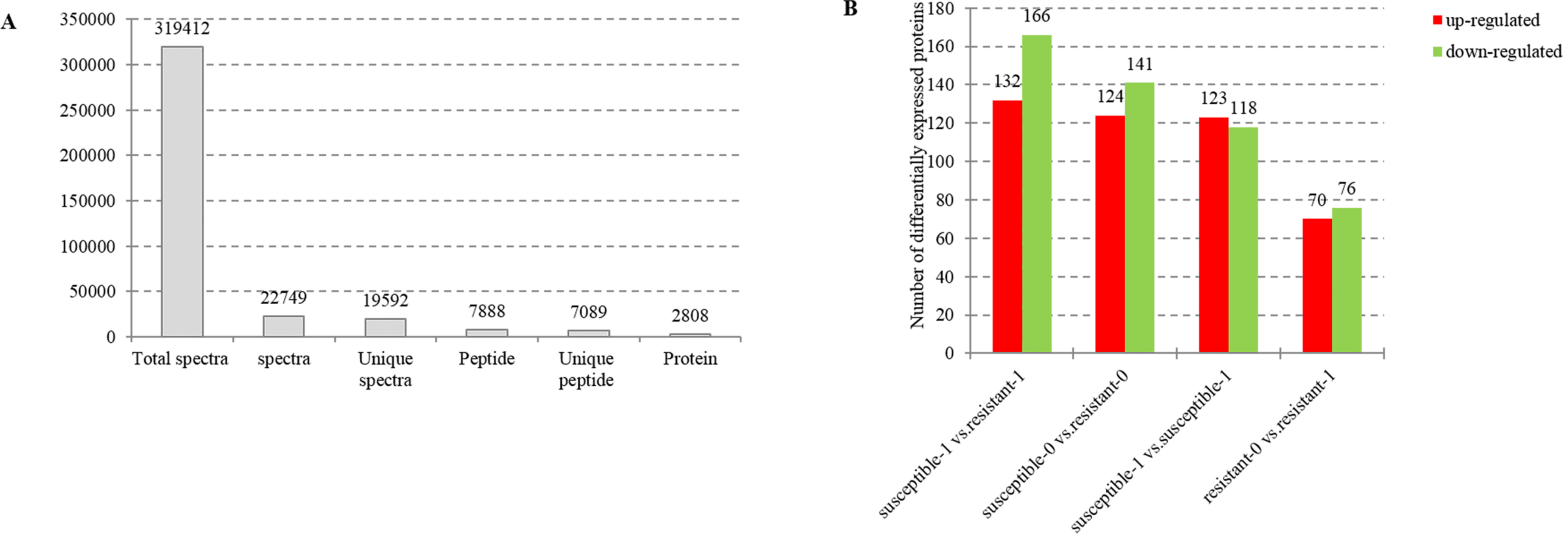
**Statistics of total spectra, unique peptides and proteins in *L*. *multiflorum* proteome (A) and effects of drought stress on the accumulation of differentially accumulated proteins (B) of two *L*. *multiflorum* lines**. The number 0 and 1 respectively reveal two Italian ryegrass lines subjected to well-water condition and naturally air drying for two hours. The red columns and green columns shows those proteins with significantly up-regulated or down-regulated accumulation.

**Fig 3:**
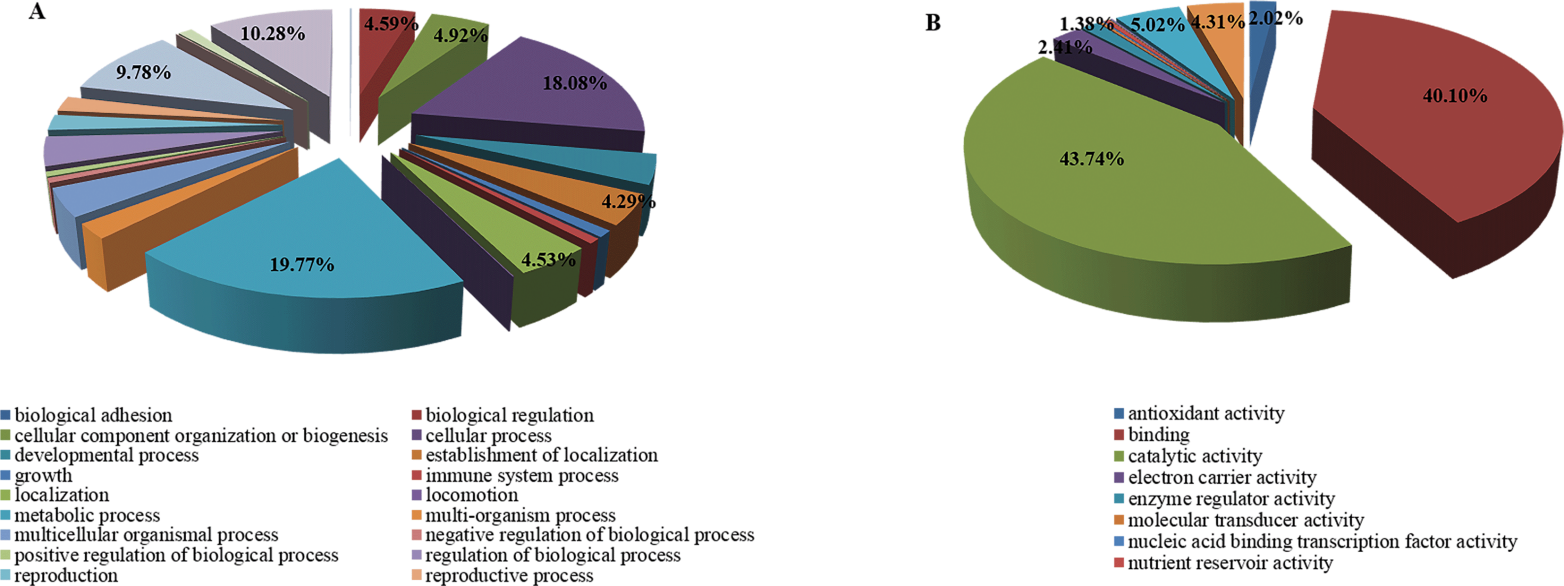
The percent of differentially accumulated proteins (DEPs) involved in biological process (A) and molecular function (B) in *L*. *multiflorum* lines.

Drought stress-induced changes in the proteome of the two *L*. *multiflorum* lines were captured by analyzing quantitative information generated by iTRAQ-based quantitative analysis and LC-MS/MS method. A total of 7, 089 unique peptides matching to 2, 808 proteins were identified with a Mascot probability analysis (Fig 2A). A differentially accumulated analysis revealed that 449 proteins were up-regulated and 501 were down-regulated (Fig 2B**)**. These proteins were categorized into biological process and molecular function based on blast2 GO program (Fig 3). The drought-regulated proteins were primarily related to metabolic process, cellular process, single-organism process and response to stimulus, which play a role in regulating the catalytic activity, structural molecule activity, transporter activity, electron carrier activity and antioxidant activity (Table 1).

**Table 1:** The detailed information of fifty-one drought-induced proteins identified from two *Lolium multiflorum* lines.

Accession.	Protein name	115/117	119/121	GO Biological process
***Tolerant (up) / Susceptible (up)***				
Egi|92429455	isocitrate lyase	2.4	5.2	tricarboxylicacidcycle/glyoxylatecycle
Cgi|39654150	β-D-Glucan Glucohydrolase	1.1	2.2	carbohydratemetabolicprocess
Pgi|262217337	cathepsin B	5.0	7.2	proteolysis/regulation of catalytic activity
Cgi|162462658	α-amylase precursor	1.5	2.5	starchcatabolicprocess/sucrosecatabolicprocess
Cgi|269316344	α-glucosidase	1.0	1.4	carbohydratemetabolicprocess
Cgi|326503406	predicted protein	1.5	4.9	hexosemetabolicprocess
Cgi|1718236	(1,4)-β-xylan endohydrolase	3.7	3.7	xylancatabolicprocess
Tgi|149392357	Predicted protein	1.0	1.5	glycolysis/gluconeogenesis/response to salt stress/glucosinolate biosynthetic process
Pgi|326533328	predicted protein	1.0	1.0	protein refolding/chloroplast organization/embryo development
Pgi|262360187	cysteine proteinase	7.0	4.6	proteolysis
gi|393450	β-amylase	2.1	5.2	polysaccharidecatabolicprocess
Cgi|326506982	β-galactosidase	2.0	1.7	carbohydratemetabolicprocess
Fgi|414881677	LRR family protein	4.1	2.8	phosphorylation/ kinase activity
Cgi|326489563	α-L-arabinofuranosidase 2	2.3	6.5	xylancatabolicprocess/L-arabinose metabolic process
gi|326497267	Xylanase inhibitor protein 1	1.5	7.1	carbohydratemetabolicprocess
gi|413948511	Phytepsin	2.2	1.9	proteolysis/lipid metabolic process/ response to salt stress
Cgi|40363751	putative β-xylosidase	3.0	3.0	carbohydratemetabolicprocess
Egi|357126982	cytochrome b5-like	1.0	1.2	electron transport chain
gi|56130862	β-amylase	1.7	4.3	starchcatabolicprocess
gi|270311550	α-amylase isoform	3.2	7.3	sucrosecatabolicprocess/ starchcatabolicprocess/ response to abscisic acid stimulus/
gi|222618904	Aspartic proteinase	9.9	13.7	proteolysis/ response to stimulus
Agi|326490063	Serine carboxypeptidase	1.9	2.2	proteolysis
gi|215398468	globulin 3C	2.7	7.7	nutrient reservoir activity/binding
gi|115349894	fasciclin-like protein	1.6	2.0	auxin polar transport/ regulation of cell size/root morphogenesis/plant-type cell wall organization
Agi|326533014	Phospho-2-dehydro-3- deoxyheptonate aldolase 1	1.2	1.2	shikimate biosynthetic process/chorismate biosynthetic process/aromatic amino acid family biosynthetic process
gi|149391177	l-ascorbate peroxidase precursor	1.0	1.0	photosynthesis /starchbiosyntheticprocess /glucosinolate metabolic proces/ /response to oxidative stress
***Tolerant (down) / Susceptible (down)***				
Cgi|213536819	tonoplast intrinsic protein	1.0	0.9	ureatransmembranetransport/ water transport
gi|357121703	uncharacterized protein LOC100836930	0.6	0.7	electron carrier activity/cytochrome-c oxidase activity
Tgi|12651627	40S ribosomal protein S8	0.8	0.7	translation
Pgi|86439735	heat shock protein 90	0.9	0.8	unfolded protein binding/ATP binding/protein folding/response to stress
gi|357134285	putative mitochondrial 2-oxoglutarate	0.9	0.7	transmembranetransport
gi|357111020	Histone H1	0.8	0.3	DNA binding/nucleosome assembly
gi|326500094	Oxygen-evolving enhancer protein 3	0.6	0.9	photosynthesis/cellular macromolecule metabolic process/ cellular component biogenesis/primary metabolic process
gi|357138855	uncharacterized protein LOC100828601	0.6	0.9	translation
Tgi|315113285	80s ribosomal protein L36	1.0	0.7	translation
Tgi|326495694	60S ribosomal protein L	0.3	0.8	translation
Tgi|414873598	40S ribosomal protein S26	0.7	1.0	translation
Pgi|326506180	Peroxiredoxin Q	0.5	0.6	oxidation-reduction process
Ggi|375152034	hsc70-interacting protein	0.7	0.7	response to cadmium ion
Tgi|357122371	ribosome-recycling factor	0.6	1.0	pentose-phosphate shunt/ plastid translation/aromatic amino acid family biosynthetic process/dolichol biosynthetic process
Tgi|357134073	proliferation-associated protein	0.7	0.7	proteolysis/cellular process
Egi|283896798	phosphoenolpyruvate carboxylase	0.9	0.8	tricarboxylic acid cycle/oxaloacetate metabolic process/carbon fixation/ photosynthesis
***Tolerant (down) / Susceptible (up)***				
^C^gi|161897650	Probable aquaporin	1.3	0.8	Ureatransmembranetransport/ Water transport
***Tolerant (up) / Susceptible (down)***				
^E^gi|326502872	Glycerate dehydrogenase	1.1	0.8	Oxidation-reductionprocess
^P^gi|115444771	peroxiredoxin 2	0.9	1.2	Oxidation-reductionprocess
^P^gi|375152308	peroxiredoxin 5	0.8	1.3	Oxidation-reductionprocess
^P^gi|326504940	predicted protein	1.0	1.2	Cysteine biosynthetic process
^C^gi|357145851	glucose-1-phosphate	0.9	1.8	Starchbiosyntheticprocess/Glycogenbiosyntheticprocess
^P^gi|375152246	dehydroascorbate reductase	0.9	1.3	Response to cyclopentenone
gi|82780752	lipid transfer protein	0.7	1.8	Lipid transport

The 115/117 and 119/121 were the fold change of well-watered and drought-treated tolerant and susceptible plants, respectively. The COG category: c Carbohydrate transport and metabolism; E Energy production conversion; A Amino acid transport and metabolism; P Posttranslational modification, protein turnover, chaperones; T Translation, ribosomal structure and biogenesis; G General function prediction only.

### Identification of proteins differentially accumulated in the two *L*. *multiflorum* Lines response to drought stress

A total of 51 drought-induced proteins were obtained from both tolerant and susceptible *L*. *multiflorum* proteomes (Table 1). Of them, 27 up-regulated and 16 down-regulated proteins having the same change trends were observed in the two *L*. *multiflorum* lines, of which up-regulated proteins associated with carbohydrate metabolism and proteolysis, while down-regulated proteins mostly participated in translation and transmembrane transport. Based on COG categories, the majority of shared proteins participated in regulation of carbohydrate transport and metabolism, post translational modification, protein turnover, translation, chaperones, ribosomal structure and biogenesis, and energy production and conversion. Comparison of the differentially accumulated proteins in the two lines identified eight responsive proteins having an opposite trend in the tolerant and susceptible lines, among them, three proteins as the important antioxidative enzymes, peroxiredoxin 2, peroxiredoxin 5 and dehydroascorbate reductase involved in oxidation-reduction processes were specifically identified in the tolerant lines. Other proteins, glycerate dehydrogenase (GDH), dehydroascorbate reductases (DHAR), glucose-1-phosphate (G1P) and lipid transfer protein (LTP), were also only up-regulated in the tolerant lines, but not susceptible line. The results not only revealed that drought tolerance of Italian ryegrass had a direct link to the specific proteins induced by drought stress, but also provided evidence that carbohydrate metabolism, oxidation-reduction processes, proteolysis, and transmembrane transport had a significant relationship with drought tolerance of Italian ryegrass.

### Network analysis for drought responsive proteins

**Fig 4:**
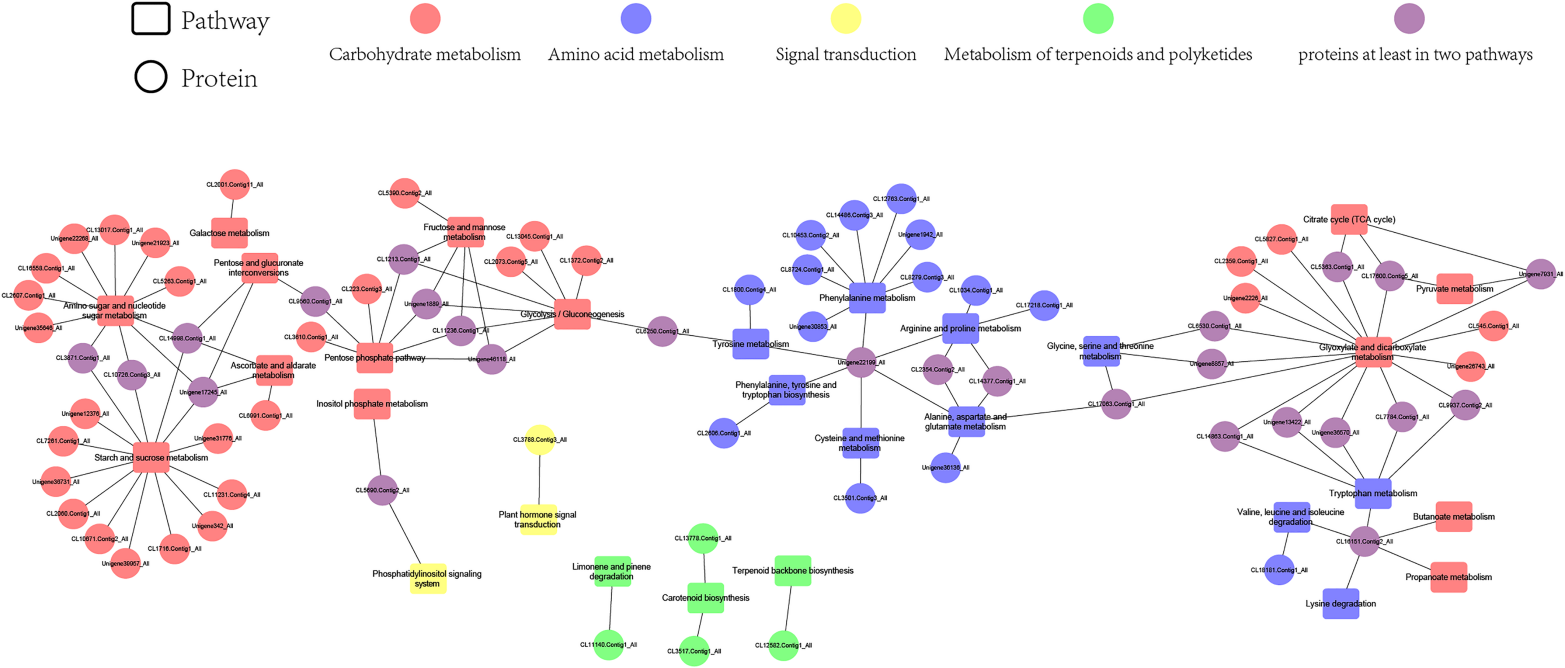
Interaction network of differentially accumulated proteins.

Proteins in plant cells and subcellular fractions play interrelated roles together in the context of networks [19]. Within the differentially accumulated proteins (susceptible vs. tolerant), a total of 76 proteins were identified using the Cytoscape software that can be mapped onto an interaction network (Table 2; Fig 4). The left region showed that responsive proteins involved in carbohydrate metabolism-related pathways, e.g. fructose and mannose metabolism, glycolysis and gluconeogenesis, citrate cycle (TCA cycle), starch and sucrose metabolism, fructose and mannose metabolism, pentose and glucuronate interconversions, galactose metabolism, pointing to a potential importance of carbohydrates in modulating the homeostasis of the drought response of Italian ryegrass. The middle part revealed that drought-related proteins enriched in nine amino acid metabolic pathways including tyrosine metabolism, phenylalanine, tyrosine and tryptophan biosynthesis, cysteine and methionine metabolism, arginine and proline metabolism, alanine, aspartate and glutamate metabolism, glycine, serine and threonine metabolism, tryptophan metabolism, valine, leucine and isoleucine degradation. The amounts of special proteins involving both of the two pathways were observed in the right side. Interestingly, a predicted protein (CL16151.Contig2) existed in tryptophan metabolism, valine, leucine, lysine, and isoleucine degradation, and butanoate and propanoate metabolism. Furthermore, a special pathway with all the differentially accumulated protein, inositol phosphate metabolism, was only found in tolerant lines, and phosphoinositide-specific phospholipase C1 (PLC) showed obviously up-regulated in phosphatidylinositol signaling systems in tolerant lines subjected to drought condition.

**Table 2:** List of differentially accumulated proteins from the interaction network.

Protein name	Description	NCBI accession number	Sources	
***Starch and sucrose metabolism***				
Unigene12376	Starch-branching enzyme	gi|301090019	Oryza sativa Indica Group	
CL7261.Contig1	Β- glucosidase	gi|39654150	Dictyostelium discoideum	
Unigene36731	Cytosolic alpha-glucan phosphorylase	gi|229610905	Hordeum vulgare	
CL2060.Contig1	α-amylase precursor	gi|162462658	Zea mays	
CL10671.Contig2	β-glucosidase 4	gi|359828768	Triticum aestivum	
Unigene39957	β-glucosidase 8	gi|326515724	Oryza sativa subsp. japonica	
CL1716.Contig1	β-D-glucan exohydrolase	gi|1203832	Hordeum vulgare	
Unigene342	α-amylase	gi|270311550	Dactylis glomerata	
CL11231.Contig4	Sucrose synthase 1	gi|326514918	Hordeum vulgare	
Unigene31776	β-amylase	gi|393450	Secale cereale	
CL3871.Contig1	Probable β-D-xylosidase 6	gi|326491679	Arabidopsis thaliana	
CL10726.Contig3	ADP-glucose pyrophosphorylase	gi|52430025	Triticum aestivum	
CL14998.Contig1	PREDICTED: UDP-glucose 6-dehydrogenase-like	gi|357114933	Brachypodium distachyon	
Unigene17245	UDP-glucose 6-dehydrogenase	gi|108711177	Oryza sativa Japonica Group	
***Amino sugar and nucleotide sugar metabolism***				
Unigene21923	Chitinase 2	gi|326492127	Oryza sativa subsp. japonica	
Cl5263.Contig1	Xylanase inhibitor protein 1			
Unigene22268	PREDICTED: UDP-glucuronic acid decarboxylase 1-like	gi|357112854	Brachypodium distachyon	
CL16558.Contig1	Chloroplast stem-loop binding protein	gi|326531332	Arabidopsis thaliana	
CL2607.Contig1	Hypothetical protein OsJ_06514	gi|222622743	Oryza sativa Japonica Group	
CL3871.Contig1	Probable β-D-xylosidase 6	gi|326491679	Arabidopsis thaliana	
CL10726.Contig3	ADP-glucose pyrophosphorylase small subunit	gi|52430025	Triticum aestivum	
CL14998.Contig1	PREDICTED: UDP-glucose 6-dehydrogenase-like	gi|357114933	Brachypodium distachyon	
Unigene17245	UDP-glucose 6-dehydrogenase	gi|108711177	Oryza sativa Japonica Group	
Unigene35646	Endochitinase 3	gi|326492127	Arachis hypogaea	
CL13017.Contig1	Xylanase inhibitor protein 1	gi|326497267	Oryza sativa subsp. japonica	
CL3871.Contig1	Probable beta-D-xylosidase 6	gi|326491679	Arabidopsis thaliana	
CL10726.Contig3	ADP-glucose pyrophosphorylase small subunit	gi|52430025	Triticum aestivum	
CL14998.Contig1	PREDICTED: UDP-glucose 6-dehydrogenase-like	gi|357114933	Brachypodium distachyon	
Unigene17245	UDP-glucose 6-dehydrogenase	gi|108711177	Oryza sativa Japonica Group	
***Ascorbate and aldarate metabolism***				
CL6991.Contig1	Chloroplast l-ascorbate peroxidase precursor	gi|149391177	Oryza sativa Indica Group	
CL14998.Contig1	PREDICTED: UDP-glucose 6-dehydrogenase-like	gi|357114933	Brachypodium distachyon	
Unigene17245	UDP-glucose 6-dehydrogenase	gi|108711177	Oryza sativa Japonica Group	
***Pentose and glucuronate interconversions***				
CL14998.Contig1	PREDICTED: UDP-glucose 6-dehydrogenase-like	gi|357114933	Brachypodium distachyon	
Unigene17245	UDP-glucose 6-dehydrogenase	gi|108711177	Oryza sativa Japonica Group	
CL9560.Contig1	Ribulose-phosphate 3-epimerase	gi|296040829	Spartina alterniflora	
***Galactose metabolism***				
CL2001.Contig11	β-galactosidase 9	gi|357124049	Oryza sativa subsp. japonica	
***Pentose phosphate pathway***				
CL3610.Contig1	Glucose-6-phosphate dehydrogenase	gi|83267994	Triticum dicoccoides	
CL9560.Contig1	Ribulose-phosphate 3-epimerase	gi|296040829	Spartina alterniflora	
CL223.Contig3	PREDICTED: uncharacterized protein LOC100829451	gi|357118665	Brachypodium distachyon	
Unigene1889	Fructose-bisphosphate aldolase 2	gi|326499908	Pisum sativum	
CL11236.Contig1	Fructose-bisphosphate aldolase	gi|326493652	Pisum sativum	
Unigene46118	Fructose-1,6-bisphosphatase	gi|219362881	Triticum aestivum	
***Inositol phosphate metabolism/Phosphatidylinositol signaling system***				
CL5690.Contig2	Phosphoinositide-specific phospholipase C1	gi|312618322|		Triticum aestivum
***Plant hormone signal transduction***				
CL3788.Contig1	Cytochrome P450 97B1	gi|401831		Hordeum vulgare
***Fructose and mannose metabolism***				
Unigene1889	Fructose-bisphosphate aldolase 2	gi|326499908		Pisum sativum
CL11236.Contig1	Fructose-bisphosphate aldolase	gi|326493652		Pisum sativum
CL11236.Contig1	Fructose-bisphosphate aldolase	gi|326493652		Pisum sativum
Unigene46118	Fructose-1,6-bisphosphatase	gi|219362881		Triticum aestivum
CL5390.Contig2	Predicted protein	gi|326523467		Hordeum vulgare
***Glycolysis/Gluconeogenesis***				
Unigene1889	Fructose-bisphosphate aldolase 2	gi|326499908		Pisum sativum
CL11236.Contig1	Fructose-bisphosphate aldolase	gi|326493652		Pisum sativum
Unigene46118	Fructose-1,6-bisphosphatase	gi|219362881		Triticum aestivum
CL2073.Contig5	Predicted protein	gi|326503406		Hordeum vulgare
CL13045.Contig1	Cytosolic glyceraldehydes-3-phophate dehydrogenase	gi|168472723		Lolium temulentum
CL1372.Contig2	3-phosphoglycerate kinase	gi|226247069		Leymus triticoides
CL6250.Contig1	Alcohol dehydrogenase class-3	gi|357137596		Oryza sativa subsp. japonica
***Tyrosine metabolism***				
CL6250.Contig1	Alcohol dehydrogenase class-3	gi|357137596		Oryza sativa subsp. japonica
CL1800.Contig4	Polyphenol oxidase	gi|46946548|		Triticum aestivum
Unigene22199	Aspartate aminotransferase	gi|165874483		Oryza granulata
***Phenylalanine*, *tyrosine and tryptophan biosynthesis***				
Unigene22199	Aspartate aminotransferase	gi|165874483		Oryza granulata
CL2606.Contig1	Phospho-2-dehydro-3-deoxyheptonate aldolase 1	gi|357148189		Brachypodium distachyon
***Cysteine and methionine metabolism***				
Unigene22199	Aspartate aminotransferase	gi|165874483		Oryza granulata
CL3501.Contig3	Cobalamin-independent methionine synthase	gi|115589740		Triticum monococcum
***Phenylalanine metabolism***				
Unigene30853	Peroxidase 1	gi|326508456		Oryza sativa subsp. japonica
CL8724.Contig1	Cationic peroxidase SPC4	gi|357166838		Sorghum bicolor
CL10453.Contig2	Peroxidase 1	gi|326518626		Zea mays
CL14486.Contig3	Peroxidase 12	gi|5777628		Arabidopsis thaliana
CL12763.Contig1	peroxidase POC1	gi|8901180		Oryza sativa Indica Group
Unigene1942	Peroxidase 2	gi|125550742		Zea mays
Unigene22199	Aspartate aminotransferase	gi|165874483		Oryza granulata
***Arginine and proline metabolism***				
CL1034.Contig1	Acetylornithine aminotransferase	gi|326510053		Alnus glutinosa
CL17218.Contig1	PREDICTED: acetylornithine deacetylase-like	gi|357137096		Brachypodium distachyon
Unigene22199	Aspartate aminotransferase	gi|165874483		Oryza granulata
CL2354.Contig2	delta-1-pyrroline-5-carboxylate dehydrogenase	gi|73913053		Triticum aestivum
CL14377.Contig1	GdhA protein	gi|129920003		Triticum durum
***Alanine*, *aspartate and glutamate metabolism***				
CL2354.Contig2	delta-1-pyrroline-5-carboxylate dehydrogenase	gi|73913053		Triticum aestivum
CL14377.Contig1	GdhA protein	gi|129920003		Triticum durum
Unigene36136	glutamine-dependent asparagine synthetase	gi|53680379		Triticum aestivum
Unigene22199	Aspartate aminotransferase	gi|165874483		Oryza granulata
CL17063.Contig1	Glutamate—glyoxylate aminotransferase 1	gi|357111762		Arabidopsis thaliana
***Glycine*, *serine and threonine metabolism***				
CL17063.Contig1	Glutamate—glyoxylate aminotransferase 1	gi|357111762		Arabidopsis thaliana
Unigene8857	Serine hydroxymethyltransferase	gi|375152224		Lolium perenne
CL6530.Contig1	Glycerate dehydrogenase	gi|326502872		Cucumis sativus
***Glyoxylate and dicarboxylate metabolism***				
Unigene8857	serine hydroxymethyltransferase	gi|375152224		Lolium perenne
CL6530.Contig1	Glycerate dehydrogenase	gi|326502872		Cucumis sativus
Unigene2226	Glycine cleavage system H protein,	gi|326505670		Oryza sativa subsp. japonica
CL2359.Contig1	Isocitrate lyase	gi|92429455		Lolium perenne
CL5827.Contig1	Peroxisomal (S)-2-hydroxy-acid oxidase GLO1	gi|357112622		Oryza sativa subsp. indica
CL5363.Contig1	Predicted protein	gi|326523589		Hordeum vulgare subsp. vulgare
CL17600.Contig5	Malate dehydrogenase	gi|13517921		Lolium perenne
CL14863.Contig1	Catalase	gi|90264977		Festuca arundinacea
Unigene13422	Catalase isozyme 2	gi|326516518		Hordeum vulgare
Unigene36570	Catalase	gi|90264977		Festuca arundinacea
CL7784.Contig1	Catalase-1	gi|2493543		Triticum aestivum
CL9937.Contig2	Catalase isozyme 1	gi|238802280		Hordeum vulgare
Unigene26743	Peroxisomal (S)-2-hydroxy-acid oxidase GLO5	gi|357111705		Oryza sativa subsp. japonica
CL545.Contig1	60S ribosomal protein L18a	gi|357132550		Oryza sativa subsp. japonica
Unigene7931	Malate dehydrogenase	gi|357147942		Arabidopsis thaliana
***Pyruvate metabolism***				
CL17600.Contig5	Malate dehydrogenase	gi|13517921		Lolium perenne
Unigene7931	Malate dehydrogenase	gi|357147942		Arabidopsis thaliana
***Citrate cycle (TCA cycle)***				
CL5363.Contig1	Predicted protein	gi|326523589		Hordeum vulgare subsp. vulgare
CL17600.Contig5	Malate dehydrogenase	gi|13517921		Lolium perenne
Unigene7931	Malate dehydrogenase	gi|357147942		Arabidopsis thaliana
***Tryptophan metabolism***				
CL14863.Contig1	Catalase	gi|90264977		Festuca arundinacea
Unigene13422	Catalase isozyme 2	gi|326516518		Hordeum vulgare
Unigene36570	catalase	gi|90264977		Festuca arundinacea
CL7784.Contig1	Catalase-1	gi|2493543		Triticum aestivum
CL9937.Contig2	Catalase isozyme 1	gi|238802280		Hordeum vulgare
CL16151.Contig2	Predicted protein	gi|326528605		Hordeum vulgare
***Valine*, *leucine and isoleucine degradation***				
CL16151.Contig2	Predicted protein	gi|326528605		Hordeum vulgare
CL18181.Contig1	Predicted protein	gi|326513882		Hordeum vulgare subsp. vulgare
***Lysine degradation/Propanoate metabolism/Butanoate metabolism***				
CL16151.Contig2	Predicted protein	gi|326528605		Hordeum vulgare
***Limonene and pinene degradation***				
CL11140.Contig1	Indole-2-monooxygenase	gi|13661750		Zea mays
***Carotenoid biosynthesis***				
CL3517.Contig1	GDSL esterase/lipase	gi|326516774		Arabidopsis thaliana
CL13778.Contig1	Cytochrome P450 97B1	gi|194699820		Pisum sativum
***Terpenoid backbone biosynthesis***				
CL12582.Contig1	Farnesyl pyrophosphate synthase	gi|326490760		Zea mays

### Confirmation of protein abundance changes by Western blotting

**Fig 5:**
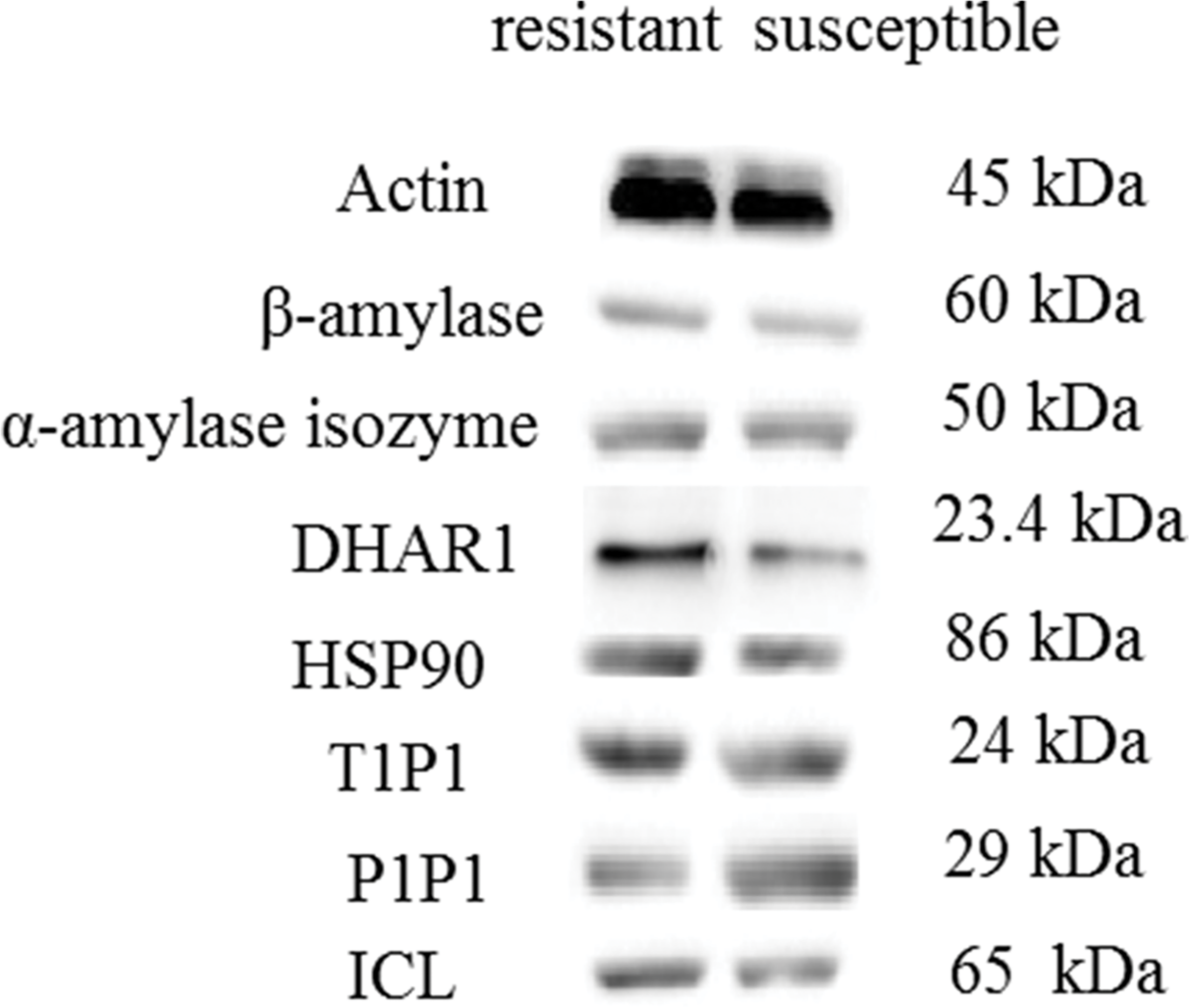
Validation of accumulation levels among differentially accumulated proteins of two *L*. *multiflorum* lines using Western blotting.

We confirmed the accumulation of seven proteins shared in the tolerant and susceptible lines by Western blotting. As shown in Fig 5, ICL (gi|92429455), α-amylase isozyme (gi|270311550), β-amylase (gi|56130862), HSP90(gi|86439735) and TIP1 (gi|213536819), showed a similar accumulation level as iTRAQ results in Table 1. Compared with drought susceptible lines, DHAR1 (gi|375152246) showed higher accumulation in tolerant lines, whereas the opposite result was observed in aquaporin, PIP1 (gi|161897650).

## Discussion

### Drought-responsive proteins related to tolerance to oxidative stress

The overproduction of reactive oxygen species (ROS) occurs when plants suffer from drought stress [20]. To prevent damage caused by ROS, plants can synthesize antioxidants, such as ascorbate, glutathione, and flavonoids, and enhance antioxidative enzymes [21]. Tolerance to abiotic stresses is involved with the ROS scavenging capacity in plants [22]. Phospholipid PHGPx is an antioxidant enzyme that directly reduces the phospholipid hydroperoxides in bio-membranes and protects cells from oxidative damage [23]. In our study, PHGPx was involved in the metabolism of arachidonic acid, which reduced the oxidative damage in the drought tolerant lines. Prx and catalase (CAT) can reduce the accumulation of ROS in plants [24, 25]. Consistent with the results of previous studies, the activity of Prx exhibited an increase trend in drought tolerant line. Dehydroascorbate reductase (DHAR) helps to enhance plant tolerance to various abiotic stresses [26]. Actually, the protective role of DHAR was found and confirmed by developing transgenic tobacco plants with cytosolic DHAR gene [27]. For Italian ryegrass, it can be speculated that DHAR has a potent protective role in defending oxidative stress. pAPX play a key role in protecting plants against oxidative stress and thus conferred abiotic stress tolerance [28]. The up-regulation of peroxisomal ascorbate peroxidase (pAPX) was observed in this study, indicating that the enzyme might reduce cell damage caused by oxidative stress in *Lolium multiflorum*. Similarly, monodehydroascorbate reductase (MDHAR) was a key enzyme in the ascorbate-glutathione cycle and served as an important antioxidant [29]. In our results, MDHAR was down-regulated in ascorbate and aldarate metabolism, but the exact mechanism is still not clear and needs further study.

### Metabolism-related proteins contributed to enhanced drought tolerance

**Fig 6:**
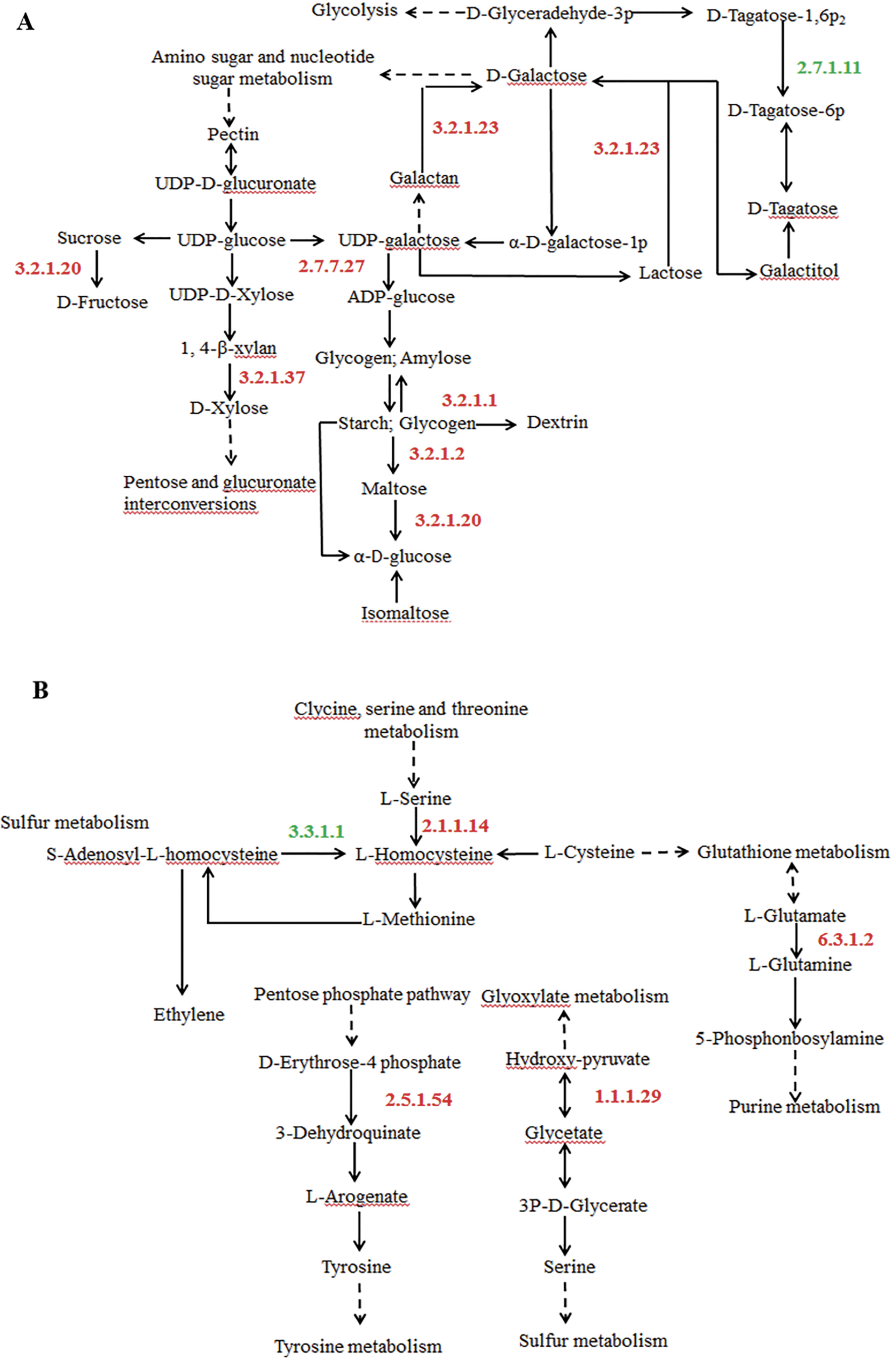
Drought stress induced proteins accumulation associating with carbohydrate metabolism (A) and amino acid metabolism (B) in tolerant *L*. *multiflorum* lines.

The levels of the readily metabolizable carbohydrates significantly increased in plants under drought stress to maintain metabolic homeostasis [30]. In this study, changes in the accumulation levels of a number of carbohydrate metabolism-associated proteins in drought tolerant lines were observed (Fig 6A). The α-glucosidase (EC 3.2.1.20) played role in catalyzing the liberation of α-D-glucose from the non-reducing end of polysaccharides [31], especially the Type I of α-glucosidase can rapidly hydrolyzes sucrose [32]. The up-regulation of regulators, such as α-glucosidase (EC 3.2.1.20) and β-galactosidase (EC 3.2.1.23), were possibly stimulated to accumulate abundant galactose in control of drought stress in Italian ryegrass. Conversely, drought stress induced a pronounced increase in the activity of enzymes that hydrolyzed starch and sucrose according to Keller et al (1993). Glucose-1-phosphate (EC 2.7.7.27), β-amylase (EC 3.2.1.2), α-amylase (EC 3.2.1.1), α-glucosidase, and putative β-D-xylosidase (EC 3.2.1.37), showed significant up-regulation and were linked with the hydrolysis of starch and sucrose under drought condition, suggesting that the drought-tolerant lines invested more carbohydrate into immediate defense against drought stress than drought susceptible lines. A putative β-D-xylosidase gene (AtBXL1) has been reported to be involved in secondary cell wall xylan synthesis [33]. Hemicelluloses were usually grouped into xylans and β-glucans [34]. The increase of the concentrations of β-D-glucan glucohydrolase (GGH) led to decrease of cell wall β-glucan concentrations in *Hordeum vulgare* [35]. In our study, down-regulated GGH not only catalyzed the hydrolytic removal of β-D-glucosyl residues [36], but also associated with enhancing drought tolerance by stimulating the degradation of β-glucans in abiotic stress.

Plant growth inhibition due to drought stress was accompanied by increased amino acid concentrations [37]. Our analysis revealed that drought-responsive proteins as regulators participated in amino acid metabolism in tolerant lines of Italian ryegrass (Fig 6B). Cobalamin-independent methionine synthase (MetE) (EC 2.1.1.14), glycerate dehydrogenase (GDH) (EC 1.1.1.29) and Phospho-2-dehydro-3-deoxyheptonate aldolase 1 (EC 2.5.1.54) were found to be up-regulated in tolerant lines, except for unnamed protein product (EC 3.3.1.1). Cobalamin-independent methionine synthase wass involved in methionine synthesis and connected sulfur and carbon metabolic networks [38]. The activity of MetE could modulate methionine biosynthesis in E.*coli* under oxidative stress conditions [39]. We observed the up-regulation of MetE specifically in tolerant lines, suggesting that this enzyme might has a positive regulatory role in methionine biosynthesis to mitigate oxidative stress. Glycerate dehydrogenase is one of the precursors of glycine betaine, which was accumulated in both plants and animals in response to drought stress [40]. Results in this study might explain that up-regulated GDH was associated with drought tolerance in Italian ryegrass. The salt-inducible cDNA had high homology to phospho-2-dehydro-3-deoxyheptonate aldolase 1 in salt-tolerant rice [41]. Our results provided evidence that phospho-2-dehydro-3-deoxyheptonate aldolase 1 as regulator contributed to drought tolerance of Italian ryegrass. It was found in previous research that proline analogues and galactose levels were higher in drought resistant varieties of potato and barley [11, 42].The higher amounts of proline involved in osmoprotection and stress signaling under drought [43]. Similar to results from other studies, we also noticed that the glutamine synthetase 1a (GS1a; EC 6.3.1.2) was up-regulated indicating that it participated in proline and glutamine synthesis to enhance drought tolerance in the tolerant line, and no change was observed in the drought susceptible lines [44–46]. A wide range of experiments showed that plants subjected to drought stress indeed expressed highconcentrations of secondary metabolites [47]. Terpenoids constitute a large and structurally diverse group of chemicals, playing diverse functional roles in plants as hormones, electron carriers and structural component of membranes [48]. Farnesyl pyrophosphate synthase (FPS) not only played a vital role in terpenoid metabolism, but also functioned as a key regulatory enzyme to control the sterol biosynthetic pathway [49]. In our study, FPS as key regulators was significantly accumulated and involved in Terpenoid backbone biosynthesis.

Inositol phosphates, diacylglycerol (DAG) and inositol 1, 4, 5-trisphosphate (IP3) assecond messengers play an important role in signal transduction pathway. Actually, the PI-PLC could mediate the production of the DAG and IP3 [50]. In our study, PI-PLC as up-regulator played role in both inositol phosphate metabolism and phosphatidylinositol signaling system in tolerant lines, indicating that PI-PLC contributed to drought-mediated abiotic stress tolerance in Italian ryegrass. Moreover, DAG as a structural lipid changed its abundance during drought condition in grasses from *Lolium-Festuca* complex was observed by Perlikowski et al. (2014) [8].

### Hydrolysis proteins and transport proteins respond to drought stress

The increase of amino acid concentration was caused by proteolysis in the advanced stages of drought [51]. Our data indicated that cathepsin B was up-regulated in the vacuole of *L*. *multiflorum* under drought stress, and might relate to the degradation of proteins. Recently, cysteine protease cathepsin B (TbCatB) was described as being involved in host protein degradation in *Trypanosoma brucei* [52]. Cysteine proteinase (CysP) showed to respond to environmental stress, such as cold or water deficiency [52] and its accumulation was observed in the leaves of tomato plants submitted to drought-stress [53]. In agreement with all the previous researches, our results indicated that CysP as an up-regulator controled protein degradation, which allowed *L*. *multiflorum* to adapt to drought stress. Aspartic proteinase (APs) are a large family of proteolytic enzymes, which are found in almost every plant [54]. APs were participated in some biological processes, e.g., stress responses, and programmed cell death [55]. We also observed the up-regulated accumulation of APs in the drought tolerant line of Italian ryegrass subjected to drought stress. This result suggested that aspartic proteinases might be important for improving drought tolerance.

Lipid transfer proteins (LTP) are small, basic, soluble proteins and are involved in stress response processes [56]. Furthermore, LTPs may repair stress-induced damage in membranes [57], and also may be responsible for increasing wax deposition [58]. In our study, LTP accumulation had dramatically up-regulated in the tolerant line, which might relate to enhance drought tolerance. Aquaporin (AQP) is plasma membrane water-transporting protein that facilitates water movement across cell membranes against osmotic gradients [59]. AQP transports water and other small molecules through biological membranes, which is vital for plants to tolerate drought [60]. According to our results, the drought tolerant line might have a lower water evaporation and higher water transport than the drought susceptible lines in drought conditions.

### Predicted (hypothetical) proteins functionally relevant to drought tolerance

The functions of hypothetical proteins are still unknown, which is a challenge not only to genome annotation but also to in depth biological interpretation [61]. Hypothetical proteins were reported previously in cereal crops under abiotic stress [12, 62]. Similarly, some predicted proteins are considered as key regulators of molecular mechanisms, with an important function in stress conditions. Kaneko et al. (1997) [63] found 3,189 predicted proteins as salt-responsive proteins in the *Synechocystis* genome (a cyanobacterium). Yang et al (2015) [64] detected 7% predicted protein that may improve drought tolerance and maintain photosynthetic activity in *Purpurea* seedlings. In our study, predicted proteins (CL16151.Contig2) played a vital role in metabolic processes of amino acids, propanoate, and butanoate. Five predicted proteins (gi|326533328, gi|149392357, gi|326503406, gi|357121703, and gi|357138855) (Table 1), were significantly accumulated in response to drought stress in two *L*. *multiflorum* lines. Based on these results, predicted proteins are valuable though with unknown functions in understanding the effects caused by complex metabolic processes.

## Conclusions

The current study revealed how drought-related proteins involved in regulatory system to adapt to abiotic stress in Italian ryegrass. Some differentially accumulated proteins, such as AtBXL1, GGH, GDH, MetE, GS1a, FPS, DAG, and PI-PLC, were found to be involved in carbohydrate metabolism, amino acid metabolism, biosynthesis of secondary metabolites, and signal transduction pathway, which might contributed to enhance drought tolerance or adaptation in *Lolium multiflorum*. The two specific metabolic processes, arachidonic acid and inositol phosphate metabolism, were differentially accumulated only in the tolerant lines. CatB, CysP, LTP and AQP were observed as drought-regulated proteins participating in hydrolysis and transmembrane transport. The activities of PHGPx, Prx, CAT, DHAR, pAPX and MDHAR associated with alleviate the accumulation of reactive oxygen species in stress inducing environments. In addition, a significant number of predicted proteins might play a vital of role in the modulation of metabolic pathways.

## Supporting information

S1 Fig Diagram of drought treatment application. (TIF)

S1 Table The detailed information of all proteins. (XLSX)
