# Supplementary figures and images for: Comparative proteomic analyses reveal the proteome response to short-term drought in Italian ryegrass (Lolium multiflorum)
Source: PLoS One. 2017 Sep 14;12(9):e0184289. doi: 10.1371/journal.pone.0184289 (PMC5598972; doi:10.1371/journal.pone.0184289)

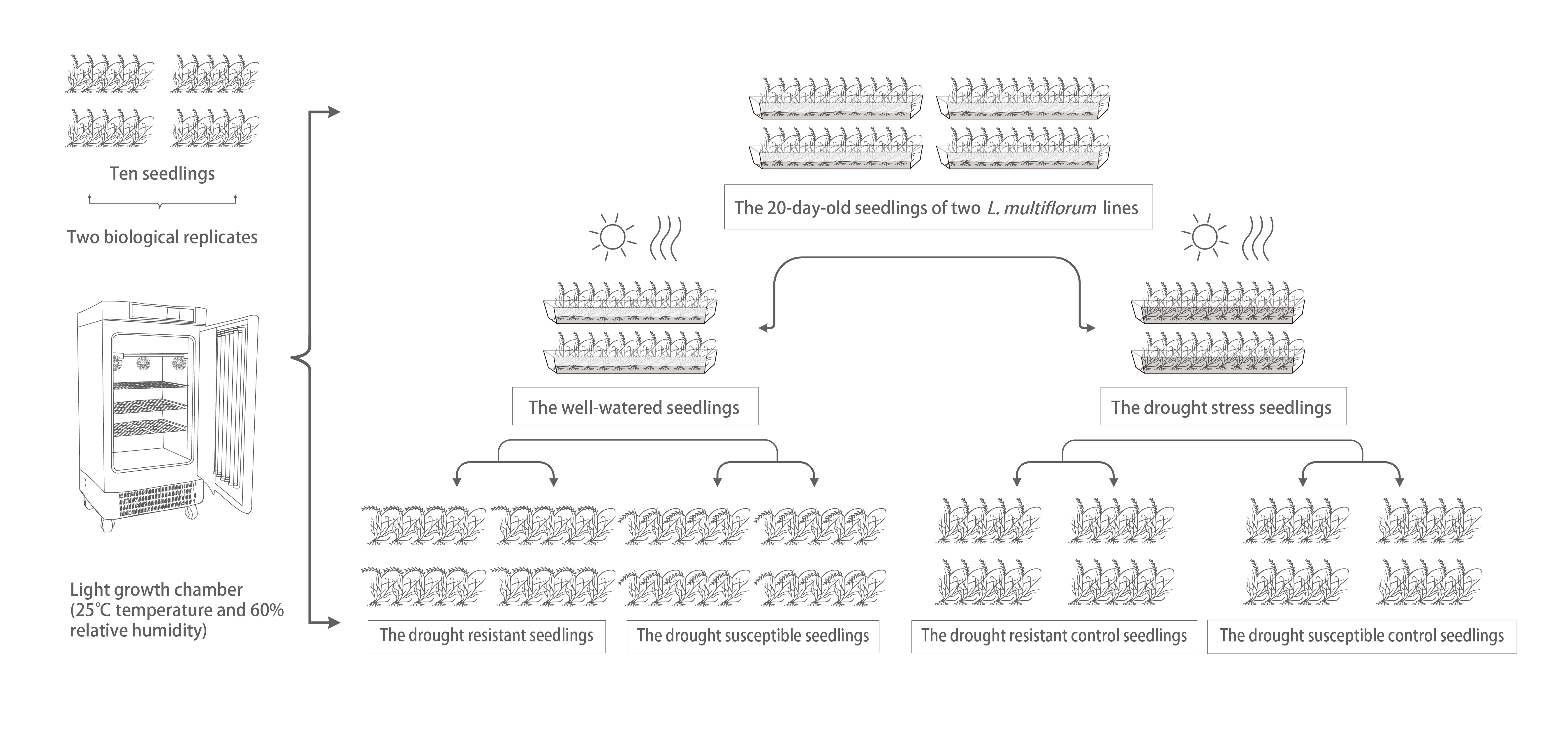

Supplement: S1 Fig — (TIF) [file pone.0184289.s001.tif]
